# Low intracranial pressure variability is associated with delayed cerebral ischemia and unfavorable outcome in aneurysmal subarachnoid hemorrhage

**DOI:** 10.1007/s10877-021-00688-y

**Published:** 2021-03-16

**Authors:** Teodor Svedung Wettervik, Timothy Howells, Anders Hånell, Elisabeth Ronne-Engström, Anders Lewén, Per Enblad

**Affiliations:** Department of Neuroscience, Section of Neurosurgery, Uppsala University, SE-751 85 Uppsala, Sweden

**Keywords:** Aneurysmal subarachnoid hemorrhage, Clinical outcome, Delayed cerebral ischemia, Intracranial pressure variability, Vasospasm

## Abstract

**Purpose:**

High intracranial pressure variability (ICPV) is associated with favorable outcome in traumatic brain injury, by mechanisms likely involving better cerebral blood flow regulation. However, less is known about ICPV in aneurysmal subarachnoid hemorrhage (aSAH). In this study, we investigated the explanatory variables for ICPV in aSAH and its association with delayed cerebral ischemia (DCI) and clinical outcome.

**Methods:**

In this retrospective study, 242 aSAH patients, treated at the neurointensive care, Uppsala, Sweden, 2008–2018, with ICP monitoring the first ten days post-ictus were included. ICPV was evaluated on three time scales: (1) ICPV-1 m—ICP slow wave amplitude of wavelengths between 55 and 15 s, (2) ICPV-30 m—the deviation from the mean ICP averaged over 30 min, and (3) ICPV-4 h—the deviation from the mean ICP averaged over 4 h. The ICPV measures were analyzed in the early phase (day 1–3), in the early vasospasm phase (day 4–6.5), and the late vasospasm phase (day 6.5–10).

**Results:**

High ICPV was associated with younger age, reduced intracranial pressure/volume reserve (high RAP), and high blood pressure variability in multiple linear regression analyses for all ICPV measures. DCI was associated with reduced ICPV in both vasospasm phases. High ICPV-1 m in the post-ictal early phase and the early vasospasm phase predicted favorable outcome in multiple logistic regressions, whereas ICPV-30 m and ICPV-4 h in the late vasospasm phase had a similar association.

**Conclusions:**

Higher ICPV may reflect more optimal cerebral vessel activity, as reduced values are associated with an increased risk of DCI and unfavorable outcome after aSAH.

## Introduction

Aneurysmal subarachnoid hemorrhage (aSAH) is a severe disease that is associated with high mortality and neurological sequele [1]. The main clinical treatment targets include early aneurysm occlusion, cerebrospinal fluid (CSF) diversion in case of acute hydrocephalus, and avoidance of delayed cerebral ischemia (DCI) [2–6]. Although intracranial pressure (ICP) is often monitored, specific treatment thresholds and theories of ICP dynamics are to a large degree based on findings in severe traumatic brain injury (TBI) [7]. There is hence a need for more studies on ICP dynamics in aSAH to better understand the specific pathophysiology in this disease.

Recently, the role of variability in various biological parameters has gained interest, both in general [8, 9] and in severe TBI [10–16]. We found in TBI that although higher ICP variability (ICPV) was associated with unfavorable variables such as higher ICP and a reduced intracranial compliance, it independently predicted favorable clinical outcome [11]. One possible explanation could be that higher ICPV reflects a healthier and more adaptive cerebrovascular system that better regulates cerebral blood flow (CBF) according to metabolic demand, resulting in reduced secondary brain injury [11]. Less is known about the physiological and prognostic meaning of ICPV in aSAH. Theoretically, a low ICPV could be associated with reduced variation of cerebral blood volume (CBV) due to increased cerebrovascular tone from vasospasm. In one study on 90 aSAH patients, Kirkness et al. evaluated mean values of ICPV and found that higher short-term ICPV predicted favorable outcome, but ICPV was not associated with cerebral vasospasm (according to transcranial Doppler) [17]. Further studies are required in this topic.

In the current study, our aim was to explore ICPV in aSAH, by studying its explanatory variables and its relations to DCI and clinical outcome. Our hypotheses were (i) higher ICPV could be explained by a reduced intracranial compliance and increased blood pressure variability, (ii) ICPV would be lower in case of DCI, and (iii) higher ICPV would be associated with favorable clinical outcome.

## Materials and methods

### Patients

Patients with aSAH admitted to the Department of Neurosurgery at the University Hospital in Uppsala, Sweden, between 2008 and 2018 were eligible for this study. Out of 605 patients with SAH and ICP monitoring, we included 242 aSAH patients aged 16 or older with ICP monitoring on all of the first 10 days post-ictus.

### Treatment protocol

Patients were treated in accordance with our standardized ICP- and CPP-oriented treatment protocol to avoid secondary insults [4, 6]. Treatment goals were ICP ≤ 20 mm Hg, CPP ≥ 60 mm Hg, systolic blood pressure > 100 mm Hg, pO_2_ > 12 kPa, arterial glucose 5–10 mmol/L (mM), electrolytes within normal ranges, slight hypervolemia with 0 fluid balance, and body temperature < 38 °C.

Patients who were unconscious (GCS M < 6) were intubated and normoventilated. Those patients were sedated with propofol and received morphine as analgesia. Neurological wake-up tests were regularly performed to detect symptoms of DCI. The patients were treated with early aneurysm occlusion, either with endovascular embolization or surgical clipping, and all patients received nimodipine. In unconscious (GCS M < 6) patients, an external ventricular drain (EVD) was inserted to monitor ICP and to drain CSF in case of high ICP. If ICP was above 20 mm Hg the EVD was opened at 15 mm Hg. In severe cases when basal ICP treatment was insufficient, thiopental coma treatment, and/or decompressive craniectomy (DC) were last-tier treatments. Arterial blood pressure (ABP) was mainly maintained with fluids. Vasopressors (preferably dobutamine, otherwise norepinephrine) were used if CPP was below 60 mm Hg and the patient did not respond to intravenous fluid treatment.

DCI was clinically defined, as new neurological deficits and/or decreased level of consciousness when other causes, e.g. hydrocephalus and hematomas, were excluded [18]. Transcranial Doppler (TCD) was not used as an adjunct for the diagnosis. If a manifest cerebral infarction was excluded, triple-H-therapy (hypertension, hypervolemia and hemodilution) including 500 ml dextran-40 solution (100 mg/ml, Meda AB) and 200 ml albumin (200 mg/ml) per day were administered for 5 days. Angioplasty was performed in case of persisting symptoms when angiography showed large-vessel vasospasm.

### Data acquisition and analyses

ICP was monitored with the EVD system (HanniSet, Xtrans, Smith Medical GmbH, Glasbrunn, Germany). ABP was measured invasively in the radial artery at heart level. Physiological data were collected at 100 Hz using the Odin software [19].

**Fig. 1:**
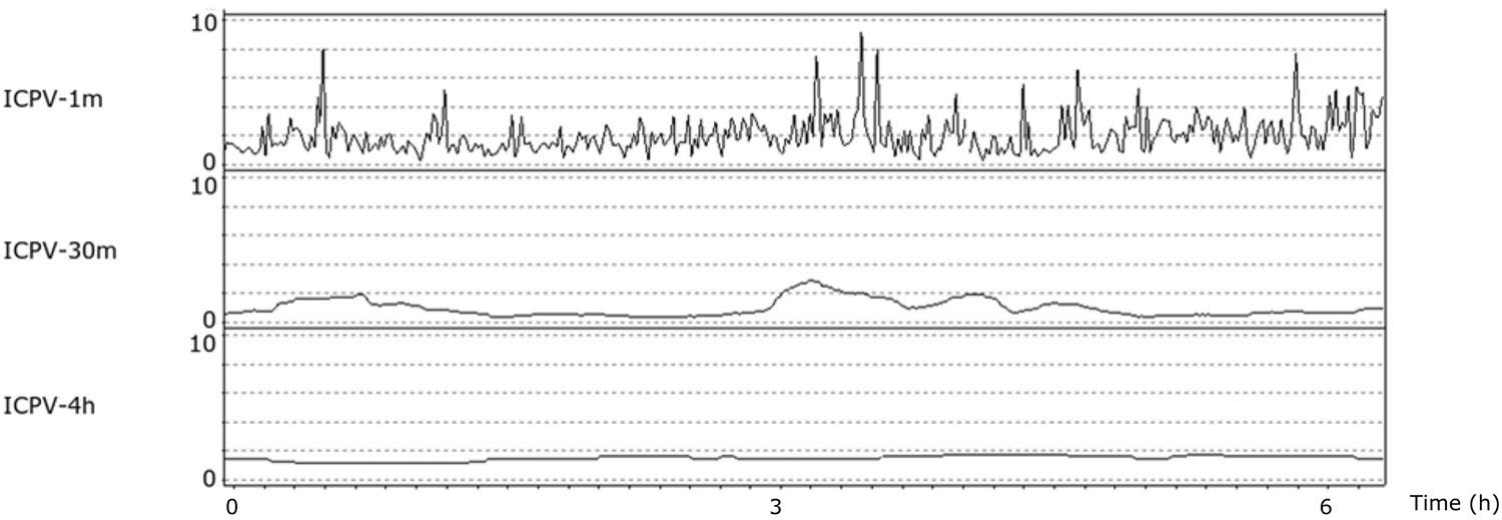
Intracranial pressure variability in one aneurysmal subarachnoid hemorrhage patient during six hours. The figure demonstrates the three ICPV measures in one aSAH patient during 6 h. The temporal variation was higher for the very short-term ICPV-1m than the more long-term ICPV-4h

ICPV was analyzed in three ways with different time intervals—(1) sub-minute interval (2) 30-min interval and (3) 4-h interval (Fig. 1) [11, 20]. In the sub-minute interval, ICPV-1 m, was calculated as the ICP slow wave amplitude with a bandpass filter, limiting the analysis to ICP oscillations with periods 55 to 15 s [20]. The second and third ICPV measures, i.e. ICPV-30 m and ICPV-4 h, were computed for every minute of monitoring as the absolute deviation from a 30-min and 4-h moving average centered on the minute, respectively [11]. Blood pressure variability (BPV) was evaluated in similar time intervals as ICPV [11, 16]. Pressure reactivity index (PRx) was calculated as the 5 min correlation of 10 s averages of ICP and MAP [21, 22]. The RAP-index (R, amplitude and pressure compliance index) was calculated as the moving 5-minute correlation between ICP amplitude and ICP, as previously described [23, 24].

### Outcome

Clinical outcome was assessed at around 12 months post-ictus, by specially trained personnel, using structured telephone interviews for the Extended Glasgow Outcome Scale (GOS-E) containing eight categories of outcome, from death to upper good recovery [25–27]. GOS-E 5–8 was considered favorable clinical outcome, whereas GOS-E 1–4 was considered unfavorable.

### Statistical analysis

The analysis aimed at finding explanatory variables for ICPV (i) and to assess the ICPV relation to DCI (ii) and clinical outcome (iii), respectively.

Nominal, ordinal, and continuous variables were described as numbers or proportions, medians [interquartile range (IQR)], and means [± standard deviation (SD)], respectively. Mean daily values for ICPV-1 m, ICPV-30 m, and ICPV-4 h were evaluated the first 10 days post-ictus for those with favorable and unfavorable outcome with 95 % confidence interval (CI). Similar calculations were done for those with and without DCI.

The 10-day period post-ictus was divided into three phases—(i) Early phase (days 1 to 3), (ii) Early vasospasm phase (days 4 to 6.5), and (iii) Late vasospasm phase (days 6.5 to 10). The vasospasm phase was hence split in the middle. Mean values for physiological variables including ICP, MAP, CPP, RAP, PRx, and the three ICPV measures were calculated for each phase. The good monitoring time (GMT %) of ICP > 20 mm Hg and CPP < 60 mm Hg were also calculated for the same phases. The thresholds were chosen in accordance with our management protocol [4]. These physiological analyses were done in the Odin software, developed at Edinburgh and Uppsala University by one of the authors (TH) [19], and the data were then transferred to SPSS version 25 (IBM Corp, Armonk, NY, USA) for further statistical analyses.

The explanatory variables for each of the three ICPV measures (outcome variable) were evaluated with the Spearman’s rank-order correlation and multiple linear regression analyses including demographic, admission, treatment, and physiological data as independent variables. We chose to focus on the late vasospasm phase for these analyses, since we found in the analysis of outcome (see below) that all three ICPV measures were associated with outcome in that phase and because this phase also represents a period with high incidence of DCI, which makes it most interesting from a clinical point of view. The treatment variables (triple-H, thiopental, and DC) in these analyses were dichotomized as yes/no, but we also took into account if they were given before/during or after the late vasospasm phase. We only considered treatment of triple-H or thiopental (serum concentration > 30 µM), respectively, as “yes” if the patient received it within the late vasospasm phase, since e.g. thiopental would not be expected to have any effect on ICPV if it had ready been eliminated to a concentration below that value. However, we considered all patients that had been operated with DC prior to, or within, the late vasospasm phase as “yes”, since the cerebral physiological effects are expected to remain until cranioplasty, which is performed much later.

The association between ICPV and DCI was evaluated, as mentioned above, with mean daily values (95 % CI) the first 10 days (DCI treatment yes/no) and with Spearman/regression analyses in the late vasospasm phase. In addition, the student’s t-test was performed for each of the three phases to analyze this association. We then only counted DCI as “yes” if they were given triple-H in that phase.

The association between ICPV and clinical outcome was analyzed separately for each ICPV measure with separate simple and multiple logistic regressions for favorable clinical outcome (GOS-E 5–8). In addition to ICPV, the multiple logistic regressions also included age, GCS M at admission, Fisher grade, GMT (%) ICP > 20 mm Hg, and GMT (%) CPP < 60 mm Hg as independent variables.

Because of the exploratory nature of the study, no adjustment for multiple testing were undertaken. A p-value < 0.05 was considered statistically significant.

### Ethics

The Uppsala regional ethical board for human research granted permission for the study.

## Results

### Demography, admission status, treatments and clinical outcome

For the 242 patients included in this study, the female/male ratio was 163/79 (67/33%) and the mean age was 58 (± 11). At admission, median GCS M was 5 (IQR 5–6), pupillary abnormalities (anisocoria/unreactive) were present in 13 (5%) patients, and the World Federation of Neurosurgical Societies (WFNS) grade was above III in 183 (76%) patients. Median Fisher grade was 4 (IQR 3–4) and the intracranial aneurysm location was in the anterior circulation in 196 (81%) patients. Endovascular aneurysm occlusion alone was done in 169 (70%) patients, surgical clip ligation in 67 (28%) patients, a combination of endovascular and surgical treatments in 2 (1%) patients, and 4 (2%) patients received no aneurysm occlusion. Sixty-one (25%) patients were treated with triple-H therapy due to DCI and the median day of treatment initiation was 4 (IQR 4–7). Twenty-four (10%) patients were treated with thiopental and the median day of treatment initiation was 3 (IQR 1–5). Thirty-four (14%) patients were treated with DC, in median on day 4 (IQR 2–7). Ten (4%) patients were treated with both thiopental and DC. Median GOS-E was 3 (IQR 3–5) and 63/179 (26%/74%) patients had favorable/unfavorable outcome.

### Systemic and cerebral physiology

The physiological variables in each of the three phases are described in Table 1. MAP and CPP gradually increased from the early phase to the late vasospasm phase. ICP gradually decreased throughout the temporal course.

**Table 1:** Systemic and cerebral physiology in three phases post-ictus

Variables	Early phase	Early vasospasm phase	Late vasospasm phase
MAP (mm Hg), mean (± SD)	89 ± 6	94 ± 8	96 ± 9
BPV-1 m (mm Hg), mean (± SD)	4.7 ± 1.4	5.1 ± 1.8	5.1 ± 1.8
BPV-30 m (mm Hg), mean (± SD)	2.6 ± 0.7	2.5 ± 0.8	2.5 ± 1.0
BPV-4 h (mm Hg), mean (± SD)	4.2 ± 1.0	4.3 ± 1.0	4.6 ± 1.3
ICP (mm Hg), mean (± SD)	12 ± 3	12 ± 3	11 ± 4
ICP > 20 mm Hg (GMT%), mean (± SD)	6 ± 8	5 ± 11	4 ± 11
ICPV-1 m (mm Hg), mean (± SD)	2.7 ± 0.9	2.6 ± 1.0	2.3 ± 1.0
ICPV-30 m (mm Hg), mean (± SD)	1.2 ± 0.3	1.1 ± 0.4	1.0 ± 0.3
ICPV-4 h (mm Hg), mean (± SD)	1.8 ± 0.4	1.7 ± 0.5	1.6 ± 0.4
RAP, mean (± SD)	0.14 ± 0.26	0.12 ± 0.22	0.12 ± 0.19
CPP (mm Hg), mean (± SD)	77 ± 7	82 ± 8	85 ± 9
CPP < 60 mm Hg (GMT%), mean (± SD)	5 ± 6	3 ± 4	2 ± 5
PRx, mean (± SD)	0.15 ± 0.13	0.16 ± 0.15	0.20 ± 0.16

### Description of ICP variability

**Fig. 2:**
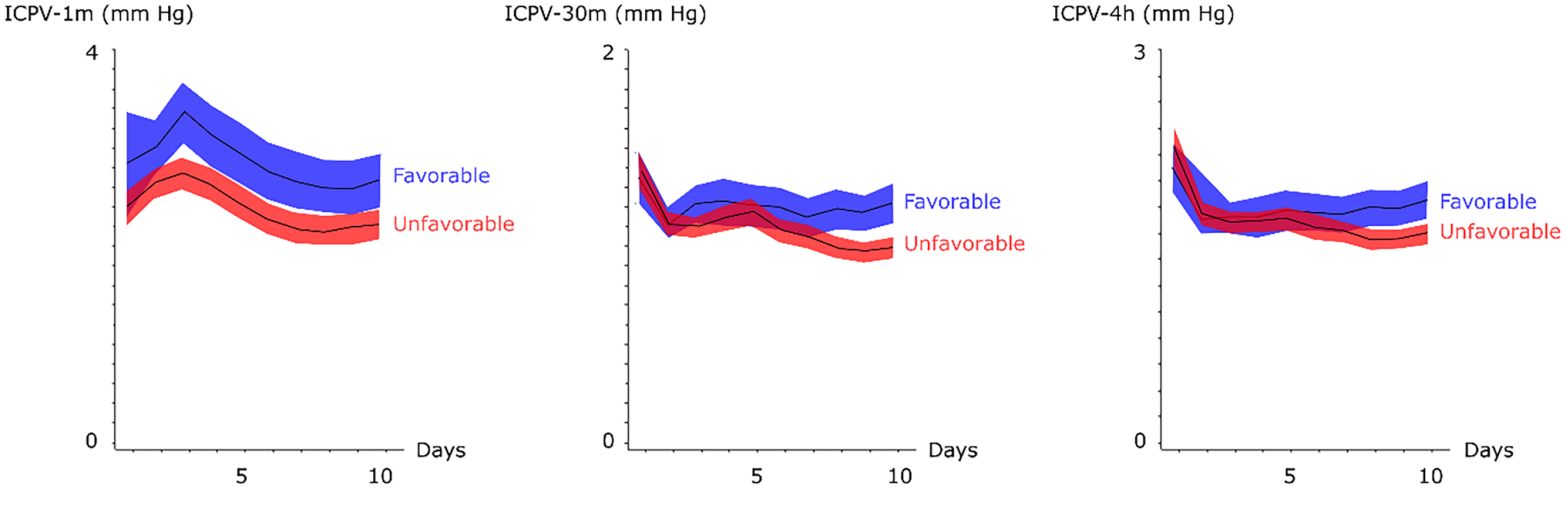
Three intracranial pressure variability measures in relation to favorable and unfavorable outcome in aneurysmal subarachnoid hemorrhage. The figure demonstrates the temporal course in the three different ICPV measures in relation to favorable (n = 63) and unfavorable (n = 179) outcome. Mean values with 95% confidence interval (CI)

Figure 1 illustrates an example of the three different ICPV measures over time. The very short-term ICPV-1 m demonstrated a greater variability than the more long-term ICPV-4 h. The ICPV measures mostly varied within the 0–10 mm Hg interval (Fig. 2; Table 1).

### ICPV: explanatory variables

Spearman correlation and multiple linear regression analyses of the explanatory variables for each ICPV measure in the late vasospasm phase are demonstrated in Table 2. In the Spearman correlation analyses, age, GCS M at admission, and Fisher grade showed no correlation with ICPV, but normal pupillary reactivity was associated with higher ICPV-1 m and ICPV-30 m. Those who were treated with thiopental in the late vasospasm phase had reductions in ICPV-1 m, but not ICPV-30 m or ICPV-4 h. DC surgery, prior to or within the late vasospasm phase, was associated with lower ICPV for all three ICPV measures. Those who received triple-H (within the late vasospasm phase) due to DCI had significantly lower ICPV. MAP and ICP were not associated with ICPV, but higher RAP, higher BPV, and lower PRx correlated with higher ICPV.

**Table 2:** Explanatory variables for ICPV in the late vasospasm phase – Spearman rank correlation and multiple linear regression analysis

Variables	ICPV-1 m				ICPV-30 m				ICPV-4 h			
	Spearman		Multiple linear regression		Spearman		Multiple linear regression		Spearman		Multiple linear regression	
	r	p-value	β	p-value	r	p-value	β	p-value	r	p-value	β	p-value
Age	− 0.02	0.75	− 0.12	***0.02***	− 0.05	0.43	− 0.20	***0.001***	− 0.01	0.87	− 0.21	***0.001***
GCS M	0.12	0.06	0.02	0.77	0.11	0.10	− 0.01	0.84	0.10	0.11	0.03	0.65
Pupil^a^	− 0.15	***0.03***	− 0.07	0.18	− 0.17	***0.01***	− 0.18	***0.02***	− 0.11	0.08	− 0.11	***0.05***
Fisher	− 0.10	0.12	0.03	0.62	− 0.09	0.15	0.01	0.84	− 0.08	0.24	0.05	0.41
Thiopental^b^	− 0.20	***0.001***	− 0.11	***0.04***	− 0.11	0.08	− 0.05	0.45	− 0.12	0.07	− 0.05	0.42
DC^c^	− 0.48	***0.001***	− 0.42	***0.001***	− 0.45	***0.001***	− 0.46	***0.001***	− 0.32	***0.001***	− 0.37	***0.001***
DCI^d^	− 0.15	***0.02***	− 0.14	***0.01***	− 0.19	***0.003***	− 0.23	***0.001***	− 0.22	***0.001***	− 0.22	***0.001***
MAP	0.08	0.21	− 0.19	***0.001***	0.01	0.99	− 0.11	0.07	− 0.07	0.29	− 0.15	***0.02***
ICP	0.06	0.36	0.22	***0.001***	0.06	0.36	0.10	0.13	− 0.09	0.18	0.03	0.68
RAP	0.20	***0.003***	0.13	***0.01***	0.29	***0.001***	0.28	***0.001***	0.50	***0.001***	0.45	***0.001***
PRx	− 0.14	***0.04***	− 0.16	***0.002***	− 0.17	***0.01***	− 0.10	0.09	− 0.14	***0.03***	− 0.06	0.25
BPV-1 m	0.41	***0.001***	0.50	***0.001***	NA	NA	NA	NA	NA	NA	NA	NA
BPV-30 m	NA	NA	NA	NA	0.22	***0.001***	0.18	***0.002***	NA	NA	NA	NA
BPV-4 h	NA	NA	NA	NA	NA	NA	NA	NA	0.15	***0.03***	0.19	***0.002***

Bold and italics indicate statistical significance

Multiple linear regression 1, ICPV-1 m, R^2^ = 0.53, ANOVA p-value = 0.001

Multiple linear regression 2, ICPV-30 m, R^2^ = 0.43, ANOVA p-value = 0.001

Multiple linear regression 3, ICPV-4 h, R^2^ = 0.44, ANOVA p-value = 0.001

^a^Pupils, 0 = normal and 1 = abnormal

^b^Thiopental, 0 = no and 1 = yes

^c^DC, 0 = no and 1 = yes

^d^DCI, 0 = no and 1 = yes

In the multiple linear regression analyses (Table 2), lower age, no DC surgery (before or within the late vasospasm phase), no DCI (triple-H within the late vasospasm phase), higher RAP, and higher BPV were independently associated with higher ICPV for all three measures. Furthermore, no thiopental (in the late vasospasm phase), lower MAP, higher ICP, and lower PRx also independently predicted higher ICPV-1 m. Normal pupillary reactivity also predicted higher ICPV-30 m and ICPV-4 h. Lower MAP was associated with higher ICPV-4 h.

### ICPV and DCI

**Fig. 3:**
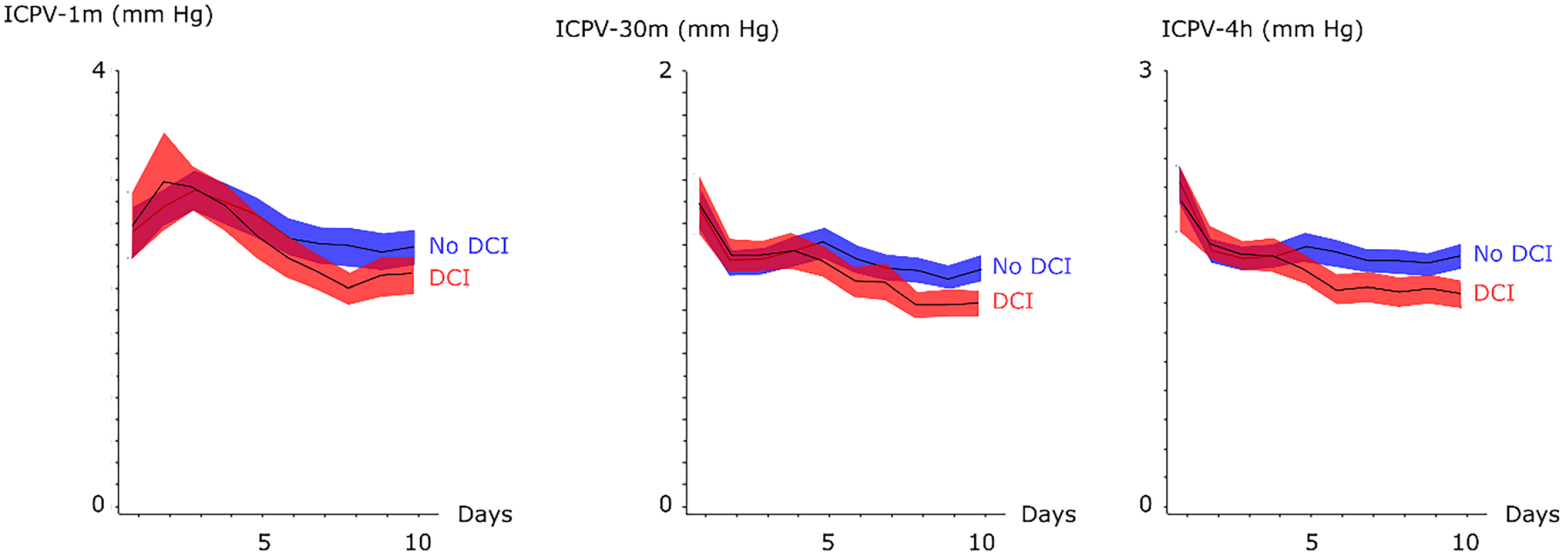
Temporal dynamics in intracranial pressure variability the first 10 days post-ictus—relation to delayed cerebral ischemia that required triple-H treatment. The figure demonstrates the temporal course in the three different ICPV measures for those that required DCI treatment with triple-H (n = 61) and those who did not (n = 181). Mean values with 95% confidence interval (CI)

Patients with DCI that required triple-H treatment had significantly lower ICPV in the vasospasm phase, but not earlier (Fig. 3). Mean values for the ICPV measures were significantly lower for those with DCI and triple-H treatment in the early vasospasm phase, including ICPV-1 m (2.3 ± 0.6 mm Hg vs. 2.6 ± 1.1 mm Hg, p-value = 0.01), ICPV-30 m (1.1 ± 0.3 mm Hg vs. 1.2 ± 0.4 mm Hg, p-value = 0.03), and ICPV-4 h (1.5 ± 0.4 mm Hg vs. 1.7 ± 0.5 mm Hg, p-value = 0.001). Similarly, mean values for the ICPV measures were significantly lower for those with DCI and triple-H treatment in the late vasospasm phase, including ICPV-1 m (2.0 ± 0.7 mm Hg vs. 2.4 ± 1.0 mm Hg, p-value = 0.004), ICPV-30 m (0.9 ± 0.3 mm Hg vs. 1.1 ± 0.4 mm Hg, p-value = 0.001) and ICP-4 h (1.5 ± 0.4 mm Hg vs. 1.7 ± 0.5 mm Hg, p-value = 0.001. There was no association between ICPV in the early phase and the risk of having DCI treated with triple-H at any time point. DCI treated with triple-H was an independent predictor of lower ICPV in the late vasospasm phase (Table 2). PRx was not significantly higher in any phase for those that developed DCI and were treated with triple-H.

### ICPV and clinical outcome

Higher ICPV-1 m was significantly associated with favorable outcome in all three phases in the simple logistic analysis (Table 3), but only in the early phase and early vasospasm phase in the multiple logistic regression analyses. Both higher ICPV-30 m and ICPV-4 h were associated with favorable outcome in simple and multiple logistic regression analyses, but only in the late vasospasm phase. In addition, younger age, higher GCS M at admission, and lower Fisher grade were associated with favorable outcome in all three phases in the multiple logistic regression analyses. Lower GMT (%) ICP > 20 mm Hg was associated with favorable outcome in the ictal phase, but not later. GMT (%) CPP < 60 mm Hg was not independently associated with outcome in any phase

**Table 3:** ICPV in relation to favorable outcome – simple and multiple logistic regression analyses for three phases post-ictus

Phase			Early phase	Early vasospasm phase	Late vasospasm phase
ICPV-1 m	Simple	OR (95 %CI)	1.63 (1.19–2.24)	1.50 (1.11–2.02)	1.48 (1.10-2.00)
		p-value	***0.003***	***0.008***	***0.01***
	Multiple	OR (95 %CI)	1.46 (1.01–2.10)	1.49 (1.06–2.10)	1.35 (0.96–1.89)
		p-value	***0.05***	***0.02***	0.09
ICPV-30 m	Simple	OR (95 %CI)	1.72 (0.69–4.24)	1.59 (0.75–3.36)	4.48 (1.88–10.69)
		p-value	0.24	0.22	***0.001***
	Multiple	OR (95 %CI)	1.93 (0.64–5.87)	1.82 (0.79–4.20)	5.38 (1.90-15.27)
		p-value	0.25	0.16	***0.002***
ICPV-4 h	Simple	OR (95 %CI)	0.99 (0.51–1.93)	1.36 (0.76–2.43)	2.82 (1.47–5.39)
		p-value	0.97	0.31	***0.002***
	Multiple	OR (95 %CI)	1.45 (0.62–3.41)	1.79 (0.92–3.50)	3.76 (1.70–8.30)
		p-value	0.39	0.09	***0.001***

Bold and italics indicate statistical significance

Multiple logistic regression analyses—age, GCS M, Fisher grade, GMT (%) ICP > 20 mm Hg and GMT (%) CPP < 60 mm Hg, in addition to each of the ICPV measures

## Discussion

In the current study of 242 aSAH patients that required ICP monitoring the first 10 days post-ictus, we found that higher ICPV, particularly in the late vasospasm phase, was associated with a lower rate of DCI and independently predicted a higher chance of favorable outcome. We suggest that higher ICPV reflects a more adaptive and active cerebrovascular system, which influences the clinical course.

### ICPV: explanatory variables

ICP variations have generated interest since the development of ICP monitoring in the NIC [28]. For example, Lundberg identified type A waves (plateau waves) with amplitudes above 50 mm Hg with a duration above 5 min at low frequencies and type B waves with smaller amplitudes around 5 mm Hg but higher frequencies around 1–2 waves/minute [29]. According to the initial theory, both of these wave types are caused by cerebral vessels reacting to unstable blood pressure, leading to variations in CBV and ICP [30, 31]. These reactions are in turn amplified when the intracranial compliance is low. Other explanations include CBF-metabolism coupling, pCO_2_-variations, and brainstem oscillations that cause secondary CBF oscillations [28, 32, 33].

In our previous study on ICPV in TBI, we found that high BPV and a reduced intracranial compliance were the main predictors of increased ICPV [11]. Similar to that study, we found that higher RAP (low intracranial compliance) and higher BPV were independently associated with higher ICPV for all three measures. We also found that lower PRx was independently associated with ICPV-1 m. Low PRx indicates intact pressure autoregulation [21, 22], which supports that higher ICPV-1 m reflects better CBF regulation. Similar correlations were found in the univariate analyses for the other two ICPV measures, but they did not hold up in the multiple regressions. It is possible that the association between ICPV-1 m and PRx was stronger because they both represent a similar time window (minutes), whereas the more long-term ICPV measures might reflect slightly different underlying mechanisms for CBV variations e.g. slower changes in cerebral metabolism. Higher age was associated with lower ICPV for all three measures. It has been suggested that aging, in general, is associated with a reduced variability in physiology [8] and when it comes to ICPV it could be a reflection of less compliant cerebral vessels and increased intracranial compliance due to cerebral atrophy. DC surgery was also independently associated with lower ICPV for all three measures, similar to our previous study [11]. This is probably a reflection of that DC drastically increases intracranial compliance so that variations in intracranial volume only lead to small changes in ICP [24]. Thiopental was associated with lower ICPV-1 m, possibly as a reflection of the metabolic suppression that led to a reduced CBV variation. Furthermore, normal pupillary status at admission independently predicted higher ICPV. As pupillary abnormalities could be related to brain herniation and brainstem injuries, this could disturb the rhythmic brainstem oscillations that might contribute to CBV variations and higher ICPV [32]. Furthermore, being treated with triple-H for DCI was also an independent significant predictor of low ICPV in the late vasospasm phase, as discussed in the next section.

In conclusion, ICPV may be explained by changes in CBV as a consequence of BPV and cerebral vessel activity. The changes in CBV are in turn amplified in a state of low intracranial compliance. The different ICPV measures also reflect their time window, as the very-short term measure might reflect more immediate myogenic vessel reflexes, whereas short- to long-term ICPV might more reflect slower cerebral metabolic processes.

### ICPV: prediction of DCI?

All three ICPV curves, particularly ICPV-1 m, demonstrated a general trend of ICPV reduction towards lower values when the patients entered the vasospasm phase. This was particularly evident for those who received triple-H due to DCI and those with unfavorable clinical outcome (Figs. 2 and 3). This is in line with that lower ICPV may reflect reductions in CBV/CBF due to cerebral vasospasm that typically occurs day 4 to 10 post-ictus. Future multimodal studies are needed to determine if lower ICPV is also associated with brain tissue hypoxia and worse cerebral energy metabolism. Kirkness et al. found no association with vasospasm (transcranial Doppler) [17], but their study was smaller and they evaluated mean values the first days post-ictus. However, we found that lower ICPV was only associated with DCI in the later course when DCI is more common, but not in the immediate phase post-ictus. This suggests that reduced ICPV was a result rather than a predictor of DCI. We have previously found that CBF increases after triple-H, but ICPV seemed to be persistently lower in the vasospasm phase despite treatment for those with DCI in the current study [34]. It is possible that although triple-H increases CBF by higher CPP and improved rheology, the cerebrovascular stiffness remains, predisposing for a persistently lower ICPV even after triple-H treatment.

The association with reduced ICPV with DCI in these patients findings may be related to the reductions in alpha variability in electroencephalography (EEG) monitoring with corresponding decreases in CBF that have been reported in cases of vasospasm/DCI in aSAH [35]. Reductions in ICPV and alpha variability may both be different representations of similar underlying mechanisms of worsening CBF regulation and cerebral energy metabolism in these patients.

We did not find any association between PRx and DCI in the current study. We have previously questioned the reliability of PRx under some circumstances after aSAH [36]. In case of severe distal small-vessel vasospasm, the capacity to autoregulate is reduced, and increases in ABP will only give small increases in ICP due to the increased cerebrovascular tone. The attenuated ICP response from increased ABP could lead to a “falsely” low (close to 0) PRx, despite severely disturbed autoregulatory status. It is hence possible that ICPV might better represent these CBF disturbances than PRx.

### ICPV: prediction of clinical outcome

Higher variability in biology, in general, indicates that the system is more healthy and adaptive [8, 9]. ICPV has gained interest in recent years, as high ICPV has been associated with favorable outcome in TBI [10–12]. This association has to some extent appeared paradoxical, since higher ICPV is associated with unfavorable variables such as a reduced intracranial compliance [11]. However, it is possible that higher ICPV may represent healthier cerebral vessels that are more compliant and active, which leads to greater variation in CBV and hence ICP.

Similar to previous TBI studies, we found that higher ICPV independently predicted favorable outcome. ICPV-1 m correlated more strongly with favorable outcome in the ictal and early vasospasm phase, whereas ICPV-30 m and ICPV-4 h only correlated with favorable outcome in the late vasospasm phase. This temporal variation in relation to clinical outcome is not completely clear, but could reflect that the mechanisms that control CBV and CBF have different time windows and that their importance differ throughout the temporal course post-ictus.

### Limitations

There are several limitations in the current study. First, the reliability of ICP wave form analysis with an open EVD has been questioned due to altering of the ICP signal [37]. However, we and others have found that the validity of ICP slow waves is preserved, which makes variables such as ICPV-1 m and PRx still valid [38, 39]. Although the ICP amplitude in the cardiac pulse wave is significantly reduced when the EVD is opened, it is still highly correlated with the ICP amplitude when the EVD is closed [39]. RAP is a continuous measure of the correlation between ICP amplitude and mean ICP over 5 min, i.e. an evaluation of relative changes rather than absolute values, and its validity therefore seems to be preserved after EVD opening [24, 39]. ICPV-30 m and ICPV-4 h evaluates the deviation of absolute ICP in relation to the average ICP for a 30-min/4-h time window. We cannot exclude that these two ICPV measures were unreliable to some extent. However, they still correlated with DCI and clinical outcome in an expected way from a biological point of view. It is possible that signal-noise was reduced when mean values were averaged over several days. Second, it is possible that the EVD management confounded our results. For example, open EVDs, particularly for low opening pressures (0–10 mm Hg), likely reduced the range in which the absolute ICP varied. It is possible that patients with a more severe brain injury required open EVD treatment to a greater extent and that DCI patients were treated with lower EVD opening pressure to improve CPP. It may therefore be difficult to determine if ICPV-30 m and ICPV-4 h reflected EVD management or cerebral vessel activity. Prospective studies are needed to truly evaluate the effect of EVD opening on these two ICPV measures. Third, TCD was not used routinely because we chose to base the judgement when triple-H treatment was indicated on the occurrence of DCI defined clinically [18]. We cannot exclude some institutional bias for when DCI and triple-H were considered. TCD could have supported presence of vasospasm but since it not excludes vasospasm with certainty we preferred to relay on the DCI diagnosis instead both in the clinical management and in the study.

## Conclusions

Higher ICPV, particularly in the vasospasm phase, correlated with a lower rate of DCI and improved clinical outcome. Higher ICPV might represent healthier cerebral vessels that are more compliant and active. Future studies are needed to determine if higher ICPV corresponds to a more favorable cerebral energy metabolic state.

## References

[1] de Rooij NK, Linn FH, van der Plas JA, Algra A, Rinkel GJ (2007). Incidence of subarachnoid haemorrhage: a systematic review with emphasis on region, age, gender and time trends. J Neurol Neurosurg Psychiatry.

[2] Diringer MN, Bleck TP, Claude Hemphill J, Menon D, Shutter L, Vespa P, Bruder N, Connolly ES, Citerio G, Gress D, Hänggi D, Hoh BL, Lanzino G, Le Roux P, Rabinstein A, Schmutzhard E, Stocchetti N, Suarez JI, Treggiari M, Tseng MY, Vergouwen MD, Wolf S, Zipfel G (2011). Critical care management of patients following aneurysmal subarachnoid hemorrhage: recommendations from the Neurocritical Care Society’s Multidisciplinary Consensus Conference. Neurocrit Care.

[3] Enblad P, Persson L (1997). Impact on clinical outcome of secondary brain insults during the neurointensive care of patients with subarachnoid haemorrhage: a pilot study. J Neurol Neurosurg Psychiatry.

[4] Ryttlefors M, Howells T, Nilsson P, Ronne-Engström E, Enblad P (2007). Secondary insults in subarachnoid hemorrhage: occurrence and impact on outcome and clinical deterioration. Neurosurgery.

[5] Connolly ES, Rabinstein AA, Carhuapoma JR, Derdeyn CP, Dion J, Higashida RT, Hoh BL, Kirkness CJ, Naidech AM, Ogilvy CS, Patel AB, Thompson BG, Vespa P (2012). Guidelines for the management of aneurysmal subarachnoid hemorrhage: a guideline for healthcare professionals from the American Heart Association/american Stroke Association. Stroke.

[6] Svedung Wettervik T, Howells T, Lewén A, Ronne-Engström E, Enblad P (2021). Temporal dynamics of ICP, CPP, PRx, and CPPopt in high-grade aneurysmal subarachnoid hemorrhage and the relation to clinical outcome. Neurocrit Care.

[7] Carney N, Totten AM, O’Reilly C, Ullman JS, Hawryluk GW, Bell MJ, Bratton SL, Chesnut R, Harris OA, Kissoon N, Rubiano AM, Shutter L, Tasker RC, Vavilala MS, Wilberger J, Wright DW, Ghajar J (2017). Guidelines for the management of severe traumatic brain injury, Fourth Edition. Neurosurgery.

[8] Lipsitz LA, Goldberger AL (1992). Loss of ‘complexity’ and aging. Potential applications of fractals and chaos theory to senescence. JAMA.

[9] Goldstein B, Fiser DH, Kelly MM, Mickelsen D, Ruttimann U, Pollack MM (1998). Decomplexification in critical illness and injury: relationship between heart rate variability, severity of illness, and outcome. Crit Care Med.

[10] Kirkness CJ, Burr RL, Mitchell PH (2008). Intracranial pressure variability and long-term outcome following traumatic brain injury. Acta Neurochir Suppl.

[11] Svedung Wettervik T, Howells T, Enblad P, Lewén A (2020). Intracranial pressure variability: relation to clinical outcome, intracranial pressure-volume index, cerebrovascular reactivity and blood pressure variability. J Clin Monit Comput.

[12] Balestreri M, Czosnyka M, Steiner LA, Schmidt E, Smielewski P, Matta B, Pickard JD (2004). Intracranial hypertension: what additional information can be derived from ICP waveform after head injury?. Acta Neurochir.

[13] Zeiler FA, Ercole A, Placek MM, Hutchinson PJ, Stocchetti N, Czosnyka M, Smieleweski P (2020). Association between physiologic signal complexity and outcomes in moderate and severe traumatic brain injury: A CENTER-TBI exploratory analysis of multiscale entropy. J Neurotrauma.

[14] Sykora M, Czosnyka M, Liu X, Donnelly J, Nasr N, Diedler J, Okoroafor F, Hutchinson P, Menon D, Smielewski P (2016). Autonomic impairment in severe traumatic brain injury: a multimodal neuromonitoring Study. Crit Care Med.

[15] Soehle M, Gies B, Smielewski P, Czosnyka M (2013). Reduced complexity of intracranial pressure observed in short time series of intracranial hypertension following traumatic brain injury in adults. J Clin Monit Comput.

[16] Svedung Wettervik T, Howells T, Lewén A, Enblad P (2020). Blood pressure variability and optimal cerebral perfusion pressure-new therapeutic targets in traumatic brain injury. Neurosurgery.

[17] Kirkness CJ, Burr RL, Mitchell PH (2009). Intracranial and blood pressure variability and long-term outcome after aneurysmal sub-arachnoid hemorrhage. Am Assoc Crit Care Nurses.

[18] Vergouwen MD, Vermeulen M, van Gijn J, Rinkel GJ, Wijdicks EF, Muizelaar JP, Mendelow AD, Juvela S, Yonas H, Terbrugge KG, Macdonald RL, Diringer MN, Broderick JP, Dreier JP, Roos YB (2010). Definition of delayed cerebral ischemia after aneurysmal subarachnoid hemorrhage as an outcome event in clinical trials and observational studies: proposal of a multidisciplinary research group. Stroke.

[19] Howells T, Elf K, Jones PA, Ronne-Engstrom E, Piper I, Nilsson P, Andrews P, Enblad P (2005). Pressure reactivity as a guide in the treatment of cerebral perfusion pressure in patients with brain trauma. J Neurosurg.

[20] Howells T, Johnson U, McKelvey T, Enblad P (2015). An optimal frequency range for assessing the pressure reactivity index in patients with traumatic brain injury. J Clin Monit Comput.

[21] Czosnyka M, Smielewski P, Kirkpatrick P, Laing RJ, Menon D, Pickard JD (1997). Continuous assessment of the cerebral vasomotor reactivity in head injury. Neurosurgery.

[22] Svedung Wettervik TM, Howells T, Enblad P, Lewén A (2018). Temporal neurophysiological dynamics in traumatic brain injury–the role of pressure reactivity and optimal cerebral perfusion pressure for predicting outcome. J Neurotrauma.

[23] Czosnyka M, Guazzo E, Whitehouse M, Smielewski P, Czosnyka Z, Kirkpatrick P, Piechnik S, Pickard JD (1996). Significance of intracranial pressure waveform analysis after head injury. Acta Neurochir.

[24] Howells T, Lewén A, Sköld MK, Ronne-Engström E, Enblad P (2012). An evaluation of three measures of intracranial compliance in traumatic brain injury patients. Intensiv Care Med.

[25] Teasdale GM, Pettigrew LE, Wilson JL, Murray G, Jennet B (1998). Analyzing outcome of treatment of severe head injury: a review and update on advancing the use of the Glasgow Outcome Scale. J Neurotrauma.

[26] Wilson JL, Pettigrew LE, Teasdale GM (1998). Structured interviews for the glasgow outcome scale and the extended glasgow outcome scale: guidelines for their use. J Neurotrauma.

[27] Nyholm L, Howells T, Enblad P, Lewén A (2013). Introduction of the Uppsala Traumatic Brain Injury register for regular surveillance of patient characteristics and neurointensive care management including secondary insult quantification and clinical outcome. Ups J Med Sci.

[28] Spiegelberg A, Preuß M, Kurtcuoglu V (2016). B-waves revisited. Interdiscip Neurosurg.

[29] Lundberg N, Troupp H, Lorin H (1965). Continuous recording of the ventricular-fluid pressure in patients with severe acute traumatic brain injury. A preliminary report. J Neurosurg.

[30] Rosner M, Miller JD, Teasdale GM (1986). The vasodilatory cascade and intracranial pressure. Intracranial pressure VI.

[31] Castellani G, Zweifel C, Kim DJ, Carrera E, Radolovich DK, Smielewski P, Hutchinson PJ, Pickard JD, Czosnyka M (2009). Plateau waves in head injured patients requiring neurocritical care. Neurocrit Care.

[32] Lang EW, Diehl RR, Timmermann L, Baron R, Deuschl G, Mehdorn HM, Zunker P (1999). Spontaneous oscillations of arterial blood pressure, cerebral and peripheral blood flow in healthy and comatose subjects. Neurol Res.

[33] Lemaire JJ, Khalil T, Cervenansky F, Gindre G, Boire JY, Bazin JE, Irthum B, Chazal J (2002). Slow pressure waves in the cranial enclosure. Acta Neurochir.

[34] Engquist H, Rostami E, Ronne-Engström E, Nilsson P, Lewén A, Enblad P (2018). Effect of HHH-therapy on regional CBF after severe subarachnoid hemorrhage studied by bedside xenon-enhanced CT. Neurocrit Care.

[35] Vespa PM, Nuwer MR, Juhász C, Alexander M, Nenov V, Martin N, Becker DP (1997). Early detection of vasospasm after acute subarachnoid hemorrhage using continuous EEG ICU monitoring. Electroencephalogr Clin Neurophysiol..

[36] Johnson U, Engquist H, Howells T, Nilsson P, Ronne-Engström E, Lewén A, Rostami E, Enblad P (2016). Bedside xenon-CT shows lower CBF in SAH patients with impaired CBF pressure autoregulation as defined by pressure reactivity index (PRx). Neurocrit Care.

[37] Hockel K, Schuhmann MU (2018). ICP monitoring by open extraventricular drainage: common practice but not suitable for advanced neuromonitoring and prone to false negativity. Acta Neurochir Suppl..

[38] Aries MJ, de Jong SF, van Dijk JM, Regtien J, Depreitere B, Czosnyka M, Smielewski P, Elting JW (2015). Observation of autoregulation indices during ventricular CSF drainage after aneurysmal subarachnoid hemorrhage: a pilot study. Neurocrit Care.

[39] Howells T, Johnson U, McKelvey T, Ronne-Engström E, Enblad P (2017). The effects of ventricular drainage on the intracranial pressure signal and the pressure reactivity index. J Clin Monit Comput..

